# Insights into the physiological, molecular, and genetic regulators of albinism in *Camellia sinensis* leaves

**DOI:** 10.3389/fgene.2023.1219335

**Published:** 2023-09-06

**Authors:** Yang Zhao, Peidi Yang, Yang Cheng, Yong Liu, Yang Yang, Zhen Liu

**Affiliations:** Tea Research Institute, Hunan Academy of Agricultural Sciences, Changsha, Hunan, China

**Keywords:** albinism, leaves, pigmentation, flavonoids, carotenoids, chlorophyll

## Abstract

**Introduction:** Yanling Yinbiancha, a cultivar of *Camellia sinensis* (L.) O. Kuntze, is an evergreen woody perennial with characteristic albino leaves. A mutant variant with green leaves on branches has been recently identified. The molecular mechanisms underlying this color variation remain unknown.

**Methods:** We aimed to utilize omics tools to decipher the molecular basis for this color variation, with the ultimate goal of enhancing existing germplasm and utilizing it in future breeding programs.

**Results and discussion:** Albinotic leaves exhibited significant chloroplast degeneration and reduced carotenoid accumulation. Transcriptomic and metabolomic analysis of the two variants revealed 1,412 differentially expressed genes and 127 differentially accumulated metabolites (DAMs). Enrichment analysis for DEGs suggested significant enrichment of pathways involved in the biosynthesis of anthocyanins, porphyrin, chlorophyll, and carotenoids. To further narrow down the causal variation for albinotic leaves, we performed a conjoint analysis of metabolome and transcriptome and identified putative candidate genes responsible for albinism in *C. sinensis* leaves. 12, 7, and 28 DEGs were significantly associated with photosynthesis, porphyrin/chlorophyll metabolism, and flavonoid metabolism, respectively. *Chlorophyllase 2*, *Chlorophyll a-Binding Protein 4A*, *Chlorophyll a-Binding Protein 24*, *Stay Green Regulator*, *Photosystem II Cytochrome b559 subunit beta along with transcription factors AP2*, *bZIP*, *MYB*, and *WRKY* were identified as a potential regulator of albinism in Yanling Yinbiancha. Moreover, we identified *Anthocyanidin reductase* and *Arabidopsis Response Regulator 1* as DEGs influencing flavonoid accumulation in albino leaves. Identification of genes related to albinism in *C. sinensis* may facilitate genetic modification or development of molecular markers, potentially enhancing cultivation efficiency and expanding the germplasm for utilization in breeding programs.

## Background

*Camellia sinensis*, commonly known as tea, is an important agricultural crop in China (Wang et al., 2018). With a long history of tea cultivation and a favorable climate for tea production, China is one of the largest producers of tea in the world (Ahmed et al., 2014). The production of tea in China covers a wide geographical range, including regions such as the southeastern coastal provinces, the central and southwestern mountainous areas, and the northern plains (Bo et al., 2012). China produces a wide variety of tea types, including green tea, black tea, oolong tea, and yellow tea, among others (Graham, 1992; Xu and Chen, 2002). The production process for each type of tea involves different methods of processing the freshly harvested tea leaves, such as steaming, pan-firing, and rolling, which results in unique flavor profiles and aromas (Han et al., 2016). In recent years, the Chinese tea industry has witnessed considerable growth, driven by increased domestic and international demand for high-quality tea products (Chen and Lu, 2021). To cater to this demand, tea producers in China have adopted new technologies and innovative techniques to enhance the quality and sustainability of tea production (Yan et al., 2020). The production of *Camellia sinensis* in China plays a significant role in the country’s agricultural economy and continues to be an important cultural and economic tradition (Xiao et al., 2018).

In plants, pigmentation is crucial for various physiological processes, including photosynthesis and protection from environmental stress (Swapnil et al., 2021; Simkin et al., 2022). Albinism in *C. sinensis* (tea) is characterized by reduced or absent pigmentation in leaves (Xie et al., 2021; Zheng et al., 2021), impacting photosynthetic performance and plant growth (Yue et al., 2021). The regulatory mechanisms behind pigment biosynthesis in plant leaves involve complex interactions between gene expression, metabolic pathways, and light signaling (Wang et al., 2014; Song et al., 2017; Li et al., 2020; Li et al., 2020; Zheng et al., 2021). However, the underlying physiological, molecular, and genetic determinants of albinism in tea plants remain poorly understood. So far, only temperature and light mediated albinism in tea plants have been explored to some extent (Ma et al., 2018; Zhang et al., 2020).

The accumulation of chlorophyll and carotenoids in albino tea leaves is controlled by genes involved in pigment biosynthesis such as tetrapyrrole pigments (e.g., chlorophylls and heme), carotenoids (e.g., lycopene and lutein), and their precursors (Qin et al., 2007; Shi et al., 2021; Wang et al., 2023), and factors like light, temperature, and hormones influence their expression (Rudiger, 2009; Folta and Carvalho, 2015; Zhang et al., 2016). Previous report suggested involvement of *STAY-GREEN* (*SGR*) and *light-harvesting complex II chlorophyll a/b binding protein* in regulating albinism in tea plants by utilizing transcriptomic approach (Ma et al., 2018). Later, Chen et al. (2022), confimed regulatory role of *SGR* by overexpression in *Arabidopsis* and *N. benthamiana*. The *SGR* overexpression accelerates the chlorophyll degradation resulting in albino or variegated phenotypes. Further research is needed to elucidate the mechanisms involved and their consequences on tea plant growth and development.

Transcriptomics and metabolomics are powerful tools for studying plant pigmentation and understanding the underlying molecular mechanisms. In this study, we employed both transcriptomics and metabolomics to investigate the albino phenomenon in the cultivar “Yanling Yinbiancha” of *C. sinensis* (L.) O. Kuntze. Our approach involved comparing leaves exhibiting albino edges with normal green leaves. By utilizing these two comprehensive analytical techniques, we aimed to gain a deeper understanding of the underlying molecular and metabolic changes associated with the albino condition in this particular cultivar. The insights gained from this study can inform the development of new strategies for improving leaf pigmentation and optimizing plant performance in *C. sinensis*.

## Materials and methods

### Plant material

Yanling Yinbiancha, with characteristic white edge on its leaves and a green central main vein, exhibits weak growth, robust cold tolerance, but it shows low tolerance to high temperatures and drought, which makes it susceptible to burning during summer. Through numerous rounds of asexual propagation and experimental cultivation since 2003, its performance traits have been established as stable. Rare instances of branch emergence and mutation resulted in the appearance of the green leaves, with the green leaves being larger than their albino counterparts and displaying a heightened growth potential in subsequent years. Yanling Yinbiancha, planted under natural conditions, leaves with albino edges and entirely green leaves were collected (one bud and two leaves were harvested on the morning of 24 April 2022) for further physiological, transcriptomic and metabolic characterization.

### Sample collection for transcriptomics and metabolomics

The tea cultivar Yanling Yinbiancha (*C. sinensis* L. O. Kuntze), exhibiting a characteristic albino leaf phenotype, was utilized in this study. Plants were grown under natural field conditions at the Tea Research Institute, Hunan Academy of Agricultural Sciences, Changsha, China. For sample collection, leaves were harvested at the one bud and two leaf stage, corresponding to the spring flush period. Specifically, the first and second fully expanded leaves along with the apical bud were collected from branches. Leaves displaying albino edges as well as entirely green leaves were sampled from separate branches of the same cultivar exhibiting these distinct phenotypes.

Sampling was conducted in the morning hours on 24 April 2022 to obtain intact RNA and metabolites. The freshly harvested leaf samples were immediately frozen in liquid nitrogen after detachment and subsequently stored at −80°C until further analysis. The sampled green and albino leaves were utilized for detailed physiological, transcriptomic and metabolomic characterization to elucidate the factors influencing the albino phenotype in this tea cultivar.

### Determination of total chlorophyll and carotenoids

The leaf samples from two genotypes were collected and weighed. 0.2 g of leaves (first and second leaf from a single node) were cut into thin strips and centrifuged using 25 mL of 95% ethanol in a 50 mL centrifuge tube. The samples were put in a dark place at room temperature for 16 h. When all the leaves turned white, absorbance was measured at 665 nm, 649 nm, and 470 nm with a UV-2102C spectrophotometer (Unico, China). Each sample has three biological replicates, and each has three technical replicates.

The calculation formulas are as follows:

Chla (mgg)=13.95A665−6.88A649×V1000W

Chlb (mgg)=24.96A665−7.32A649×V1000W

Carto (mgg)=1000A470−2.05Chla−114.8Chlb/245×V1000W

### Metabolomic profiles

Metabolic profiling was outsourced to Wuhan Metware Biotechnology Co., Ltd. (https://www.metware.cn), a well-established and renowned service provider specializing in metabolomics analysis. The meticulous execution of the metabolic profiling involved a step-by-step approach as described below:

Firstly, cryo-preserved leaf samples were carefully collected and weighed with precision, ensuring consistency in the amount of material used for analysis. These samples were specifically chosen to represent both the green and albino leaves of the tea plants, forming the basis for our comparative study. Next, metabolites were extracted from the leaf samples using 70% methanol as the solvent. Methanol extraction is a highly efficient method that allows us to retrieve a wide range of metabolites from the plant tissues. Subsequently, the methanol extracts underwent state-of-the-art liquid chromatography mass-spectrometry/M.S. analysis (LC-MS/MS) using an advanced UPLC Shim-pack UFLC SHIMADZU CBM30A system, coupled with an Applied Biosystems 6500 QTRAP mass spectrometer. LC-MS/MS is a powerful technique known for its high-throughput capabilities, sensitivity, and accuracy in detecting a diverse array of metabolites.

To identify the detected metabolites, a comprehensive approach was adopted, utilizing Metware’s extensive metabolite database in conjunction with publicly available metabolite databases. This comprehensive strategy ensured the reliable identification of metabolites based on their mass spectra and retention times. The quantification of the identified metabolites was performed based on the LC-MS/MS data. This crucial step allowed us to precisely measure the abundance of each metabolite in the samples, facilitating quantitative comparisons between the green and albino leaves. To assess the differences in metabolite accumulation patterns between the two leaf types, we employed orthogonal partial least squares discriminant analysis (OPLS-DA), a powerful multivariate statistical method. OPLS-DA aided in identifying significant changes in metabolite levels, providing valuable insights into the variations between the green and albino leaves under study.

Finally, the identification of Differentially Accumulated Metabolites (DAMs) was based on defined criteria, with metabolites displaying |Log_2_ Foldchange| ≥ 1 and VIP ≥1 considered as key indicators of differences in abundance between the green and albino leaves. These DAMs hold great significance in unraveling the metabolic changes associated with albinism in tea plants, shedding light on potential mechanisms underlying this intriguing phenotype.

### Transcriptomic profiles

Total RNA was extracted from the samples with the RNA Extraction kit (TIANGEN, Beijing, China). The RNA quality and concentration were assessed with agarose gel electrophoresis and NanoDrop2000 spectrophotometer. Quality testing, library construction, and sequencing for each sample were done at Metware Biotechnology Co., Ltd. (https://www.metware.cn), following the company’s standard procedures. Low-quality data were removed for downstream analysis, and high-quality clean reads were used for transcriptome quantification. The clean reads were localized to the reference genome (Xia et al., 2020) using Hisat2 (Sirén et al., 2014). Reads per kilobase mapping (FPKM) for all genes were used to determine gene expression values, and further screening for differentially expressed genes (DEGs) was performed. The identified DEGs were further enriched by KEGG analysis. Gene Ontology (GO) annotation and Kyoto Encyclopedia of Genes and Genomes (KEGG) pathway enrichment analysis were applied using Tbtools software (Chen et al., 2020). Heat maps were generated using the OmicStudio tools at https://www.omicstudio.cn/tool.

## Results

### Physiological parameters

**FIGURE 1:**
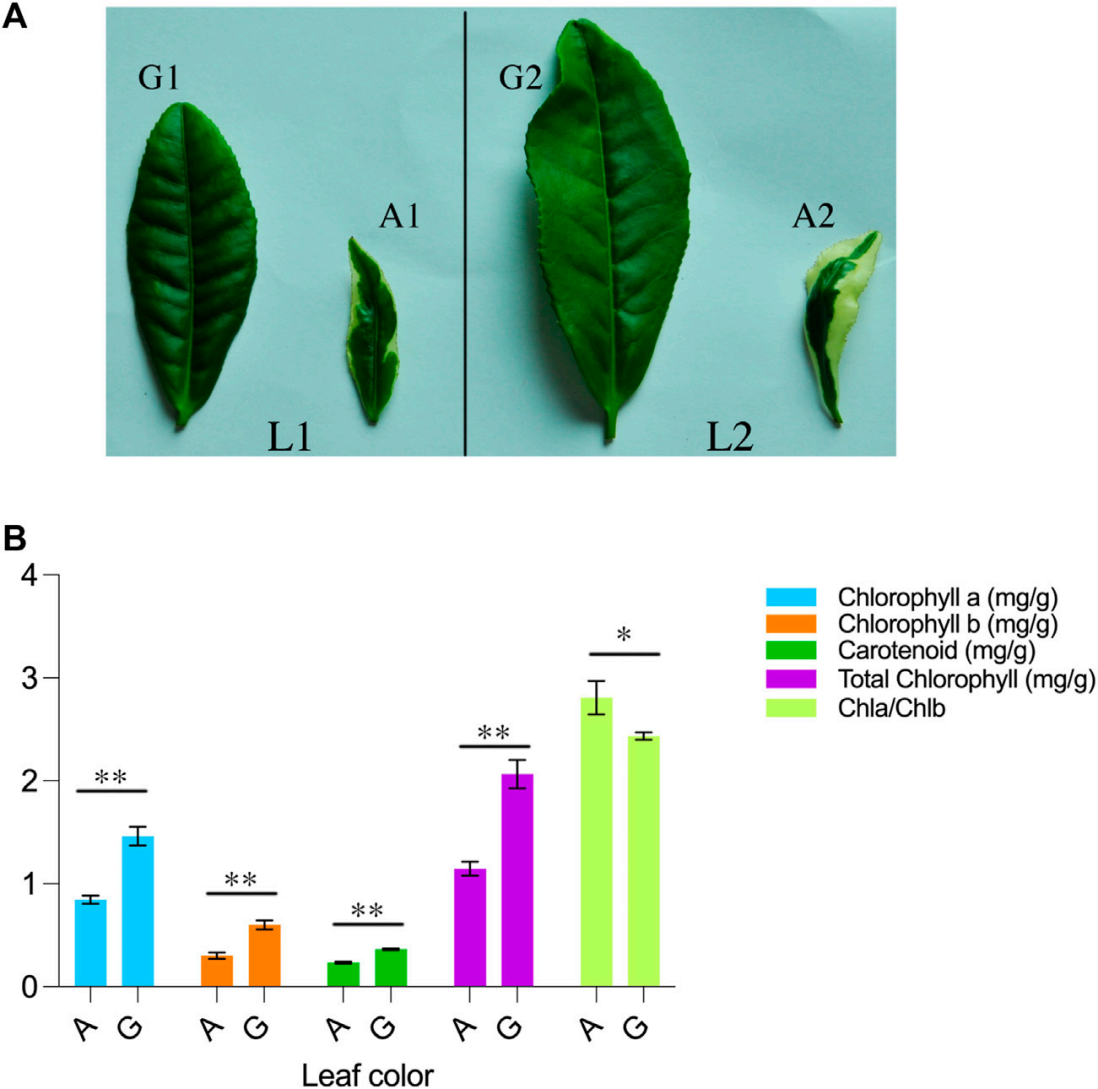
Morpho-physiological description of *C. sinensis* **(A)** A visual representation of *Camellia sinensis* leaves is provided, wherein L1 and L2 denote the first and second leaves of the plant. The first and second leaves of the green branch are designated as G1 and G2, respectively, while the first and second leaves of the mutant branch are labeled as A1 and A2 **(B)** Chlorophyll and carotenoid determination in green and albino leaves. A represents the albino leaves and G represents green leaves. ** indicate highly significant differences (*p* < 0.01) and * represents significant differences (*p* < 0.05).

Yanling Yinbiancha, a cultivar of *C. sinensis* (L.) O. Ktze, was used in this study. The Yanling Yinbiancha plant exhibits a characteristic white edge on its leaves, with a green central main vein. To further characterize this phenomenon, we estimated physiological parameters, including chlorophyll a, chlorophyll b, and carotenoids from leaf samples in Yanling Yinbiancha with albino and green leaves. The mean performance of each trait was estimated, and parameters depicted significant differences between albino and green genotypes (Figures 1A, B).

In comparing green leaves to albino leaves, there was a significant increase observed in the levels of total chlorophyll, chlorophyll a, chlorophyll b, and carotenoids in the green leaves. Specifically, the green leaves exhibited a 55% increase in total chlorophyll, a 57% increase in chlorophyll a, a 50% increase in chlorophyll b, and a 64% increase in carotenoid content compared to the albino leaves. Interestingly, the ratio of chlorophyll a to chlorophyll b (Chla/Chlb) was found to be higher in albino leaves when compared to green leaves. This suggests that albino leaves have a relatively higher proportion of chlorophyll a in relation to chlorophyll b compared to green leaves. These findings indicate that the presence of green pigmentation in leaves is associated with higher levels of total chlorophyll, chlorophyll a, chlorophyll b, and carotenoids, highlighting their role in photosynthetic processes.

### Overview of the metabolomic and transcriptomic profile of tea plant leaves

**FIGURE 2:**
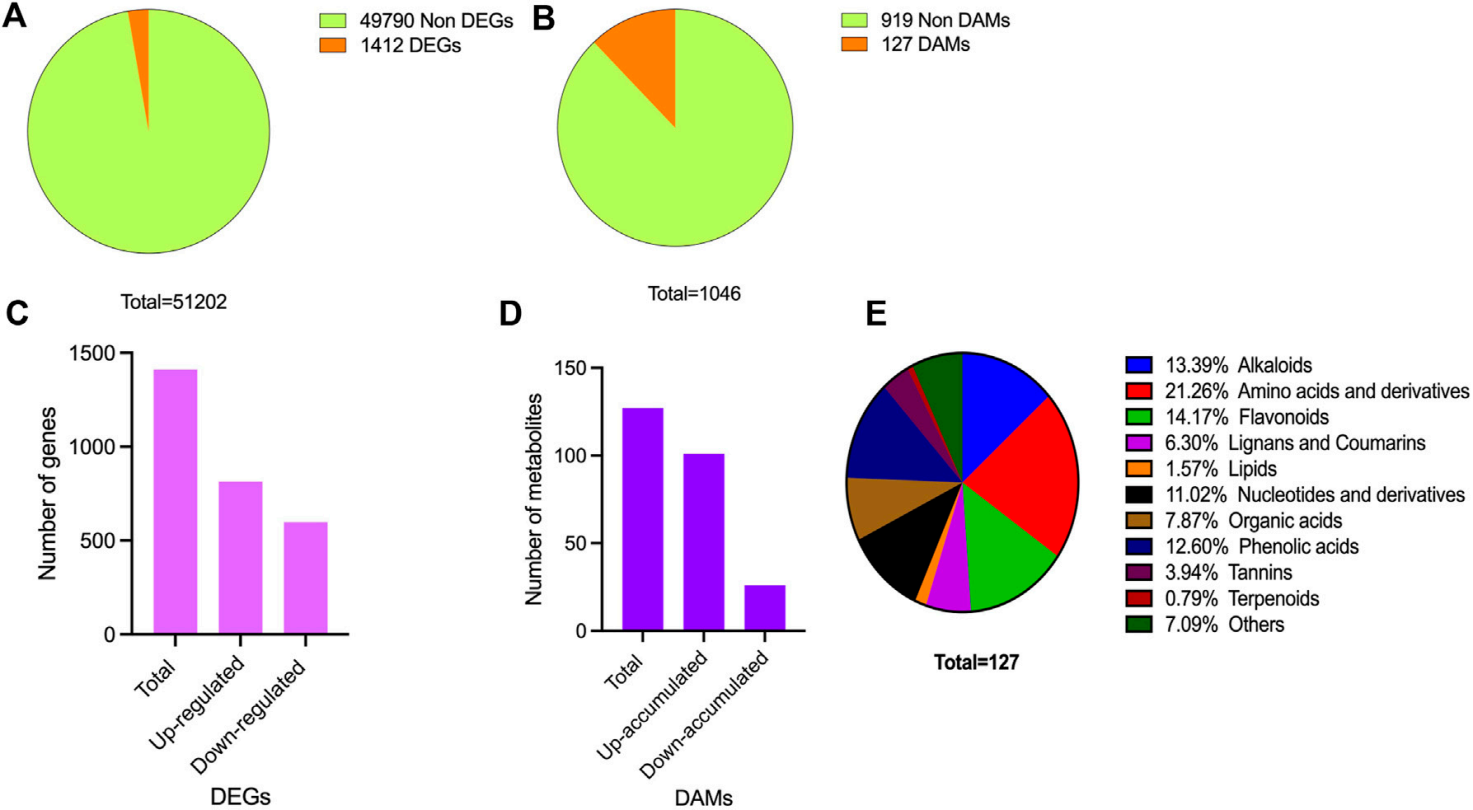
Overview of transcriptome and metabolome profile **(A)** DEGs identification **(B)** DAMs identification **(C)** DEGs characterization in upregulated and downregulated DEGs **(D)** DAMs characterization in up-accumulated and down-accumulated DAMs **(E)** Accumulation pattern of differentially accumulated metabolites and their classification.

For metabolome profiling, leaf samples from Yanling Yinbiancha and its mutant branch with green leaves were collected and subjected to subsequent analysis. Metabolome profiling resulted in the identification of 1,046 metabolites belonging to subclasses alkaloids (61), amino acids and derivatives (83), flavonoids (307), lignans and coumarins (38), lipids (89), nucleotides and derivatives (63), organic acids (67), phenolic acids (209), tannins and terpenoids (30), and others (99) (Figure 2B; Supplementary Table S1). The accumulation pattern of metabolites showed significant differences in the metabolic profile of albino and green leaves. We further identified differentially accumulated metabolites Albino vs. Green (A vs. G) comparison (127 DAMs) (Figure 2B; Supplementary Table S1). Among 127 DAMs, 101 up-accumulated and 26 down-accumulated metabolites were identified (Figure 2D). Amino acids, alkaloids, flavonoids, and phenolics were among the metabolites accumulated differentially (Figure 2E).

To gain insights into the molecular mechanisms underlying the observed differences in pigmentation between green and albino leaves, transcriptome profiling was conducted on the collected leaf samples. This technique allowed for the characterization of the expression profiles of genes that showed differential expression between the two leaf types. We constructed six libraries and FPKM-based expression profiles of 51,202 genes were quantified. A total of 42.01 Gb of data was generated. A total of 308,901,608 raw reads were obtained, and after filtering, 286,071,524 clean reads were kept (Supplementary Table S2). The quality check was performed to confer the reliability and reproducibility of the data. Q20 and Q30 were estimated to be over 96% and 91%, respectively (Supplementary Table S2). Moreover, GC contents ranged from 44.03% to 44.54%. All the transcriptome data were subjected to principal component analysis to identify the corresponding variation. PC1 and PC2 covered 35.39% and 19.49% of the variation, and replicates from each group were clustered, depicting the significance of the transcriptome data sets for downstream analysis (Supplementary Figure S1).

**FIGURE 3:**
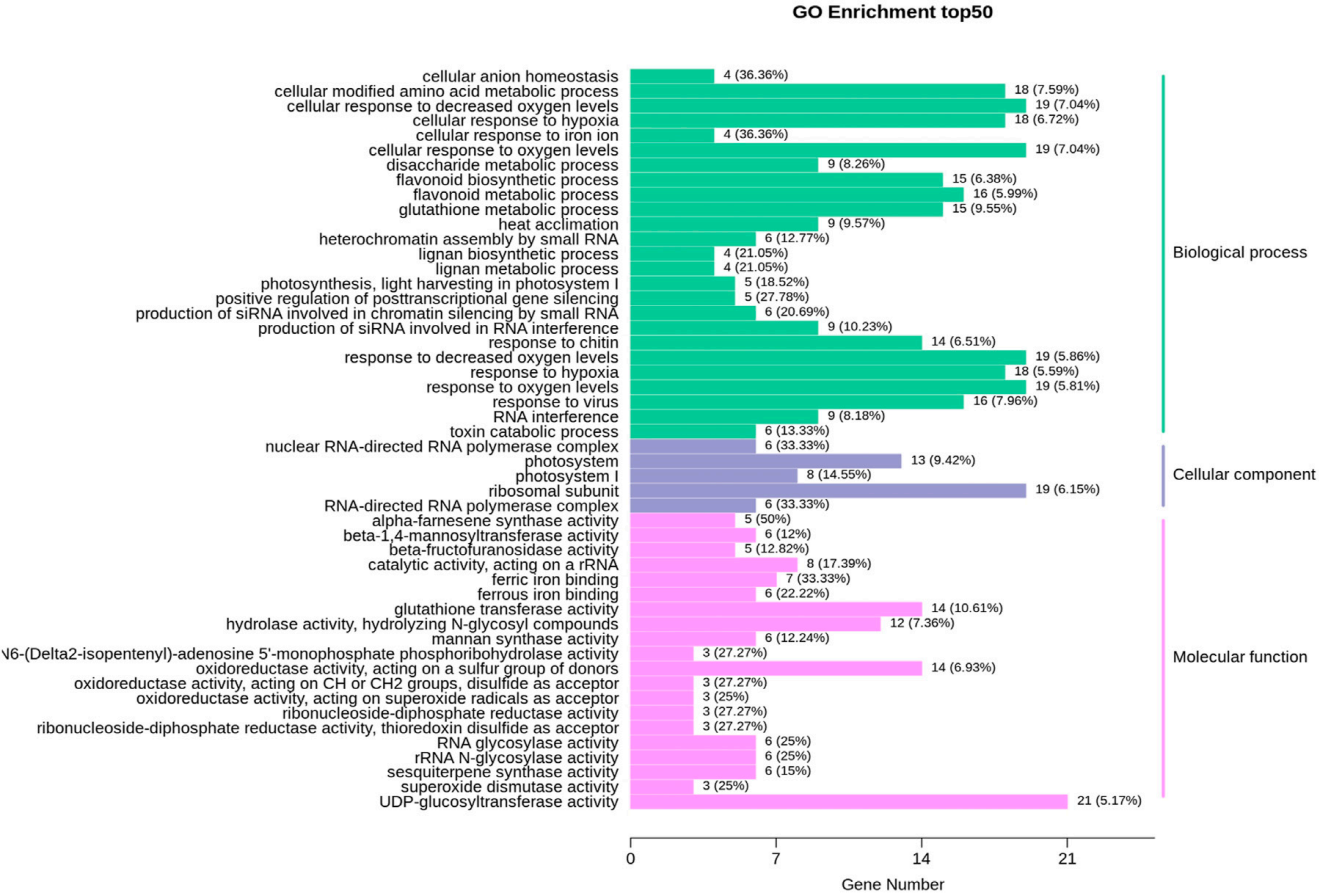
GO enrichment analysis for differentially expressed genes (DEGs) in comparison of albino leaves with green leaves (A vs. G).

The expression profile of genes in both albino and green variants were compared, and 1,412 differentially expressed genes (DEGs) were identified (Figure 2A). Among them, 814 genes were upregulated in the green variant compared to the albino, and 598 were downregulated (Figure 2C). Go enrichment analysis of DEGs suggested significant enrichment of GO terms associated with flavonoid biosynthesis, photosynthesis, light harvesting in photosystem 1, and response to oxygen levels (Figure 3).

### Factors contributing to albinism in tea plant leaves

**FIGURE 4:**
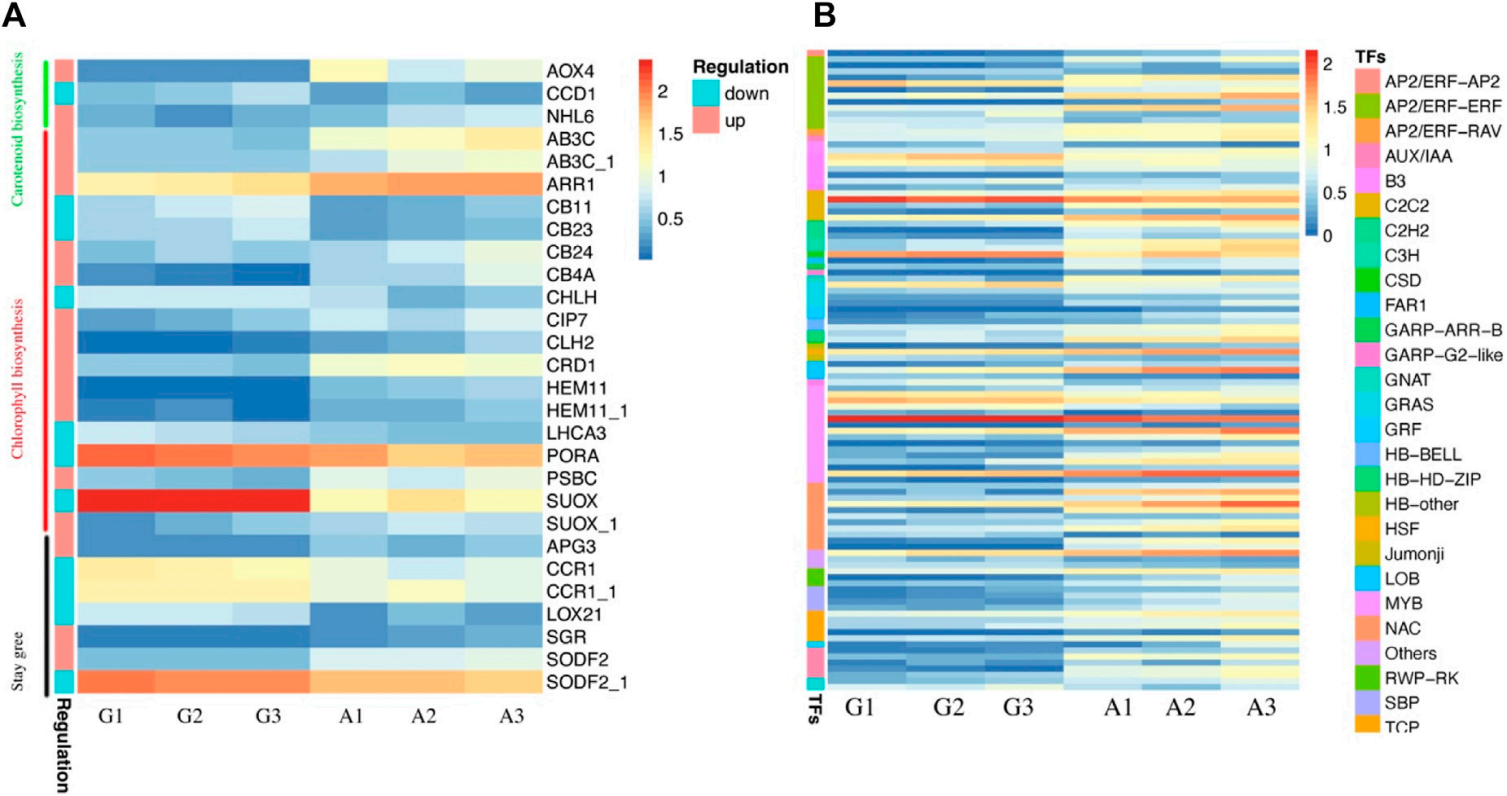
Characterization of identified DEGs and Transcription factors in green and albino leaves **(A)** DEGs identified in carotenoid biosynthesis, chlorophyll biosynthesis and stay green trait. G denotes to green leaves and A denotes to albino leaves, while 1, 2, and 3 are corresponding replicates. The regulation pattern depicting upregulation and downregulation in albino leaves compared to green leaves (A vs. G**) (B)** Expression profile of transcription factors identified as DEGs.

To comprehend the molecular basis of albinism in Yanling Yinbiancha, we compared metabolomic and transcriptomic profiles of both albino and green variants. As it is evident from previous studies that albino leaves lack chlorophyll contents considerably. Physiological data also suggested the same where we quantified total chlorophyll in both variants, and albino leaves were identified with less chlorophyll contents. Moreover, a decrease in chlorophyll contents can simultaneously result in reduced carotenoids in leaves. Therefore, we screened the DEGs and identified the DEGs associated with chlorophyll biosynthesis, carotenoid biosynthesis, and stay green trait based on the annotation information (Supplementary Table S3; Figure 4A). The genes related to chlorophyll biosynthesis, including *CLH2*, *CB4A*, *CB24*, *SGR*, *PSBC*, *ARR1*, *AB3C*, *HEM11*, *SUOX* (*TEA000669*), *CRD1*, and *CIP7,* were identified with upregulated expression pattern in albino leaves compared to green leaves. While *LHCA3*, *PORA*, *CB23*, *CB11*, *CHLH*, and *SUOX* (*TEA000535*) were identified with downregulated expression patterns in albino leaves compared to green leaves. It is worth noting that *SGR* (*protein STAY-GREEN*) was significantly suppressed in green leaves, indicating that chlorophyll biosynthesis was positively regulated in green leaves. Moreover, two genes, *NHL6* and *AOX4* (associated with carotenoid biosynthesis showed upregulated expression patterns in albino leaves, and one gene (*CCD1*) was identified with a downregulated expression pattern in albino leaves. The annotation information and expression profile of these suggested candidacy for further functional verification of these genes and their role in conferring albinism in *C. sinensis*.

Moreover, we identified 105 transcription factors as DEGs. The expression profile of TFs (81) suggested a significant increase in expression pattern in albino leaves compared to green leaves, while 23 were downregulated in albino leaves (Figure 4B). The identified transcription factors belong to *AP2, AUX, B3, bHLH, bZIP, C2C2, GARP, CSD, GRAS, GRF, MYB, NAC,* and *WRKY* families.

### Impact of albinism on flavonoid accumulation pattern

**FIGURE 5:**
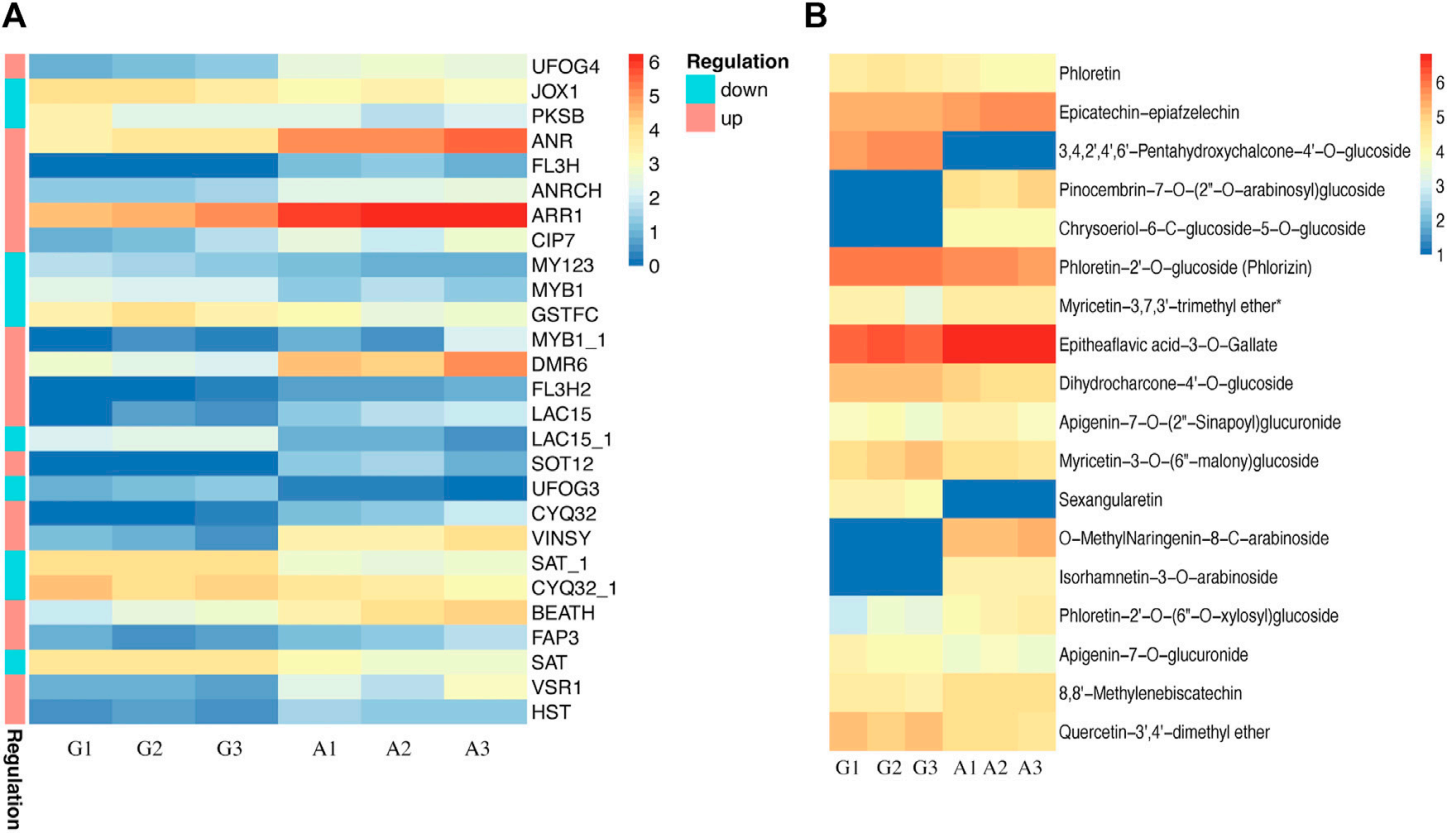
Characterization of flavonoid biosynthesis in green and albino leaves **(A)** DEGs identified in flavonoid biosynthesis. G denotes to green leaves and A denotes to albino leaves, while 1, 2, and 3 are corresponding replicates. The regulation pattern depicting upregulation and downregulation in albino leaves compared to green leaves (A vs. G**) (B)** Accumulation patterns of differentially accumulated metabolites (DAMs) in green and albino leaves. G denotes to green leaves and A denotes to albino leaves, while 1, 2, and 3 are corresponding replicates.

Previously published reports suggested an indirect relationship between albinism and flavonoid accumulation patterns. Lack of chlorophyll in albino leaves makes them vulnerable to light stress resulting in increased flavonoid accumulation in leaves. Therefore, we further explored the transcriptome and metabolome datasets to identify differentially accumulated flavonoids and DEGs associated with flavonoid biosynthesis. The most prominent DEGs associated with flavonoids biosynthesis included *ANR, ARR1, DMR6, VINSY,* and *VSR1,* showing upregulated expression patterns in albino leaves, while *JOX1, PKSB, MY123, MYB1, GSTFC, LAC15_1, UFOG3, SAT, CYQ32_1, HST* and *SAT_1* were upregulated in green leaves (Figure 5A). The differential expression profile of these genes suggested regulation of flavonoids in albino and green leaves, which was further confirmed by flavonoid accumulation patterns in both types (Figure 4B). Flavonoids including, Epicatechin-epiafzelechin, Pinocembrin-7-O-(2″-O-arabinosyl)glucoside, Chrysoeriol-6-C-glucoside-5-O-glucoside, Myricetin-3,7,3′-trimethyl ether, Epitheaflavic acid-3-O-Gallate, Apigenin-7-O-(2″-Sinapoyl) glucuronide, O-MethylNaringenin-8-C-arabinoside, Isorhamnetin-3-O-arabinoside, Phloretin-2′-O-(6″-O-xylosyl) glucoside, and 8,8′-Methylenebiscatechin were up-accumulated in albino leaves while Phloretin, 3,4,2′,4′,6′-Pentahydroxychalcone-4′-O-glucoside, Phloretin-2′-O-glucoside (Phlorizin), Dihydrocharcone-4′-O-glucoside, Myricetin-3-O-(6″-malony)glucoside, Sexangularetin, Apigenin-7-O-glucuronide, and Quercetin-3′,4′-dimethyl ether were up-accumulated in green leaves.

### Conjoint analysis of transcriptome and metabolome pertaining to tea plant

**FIGURE 6:**
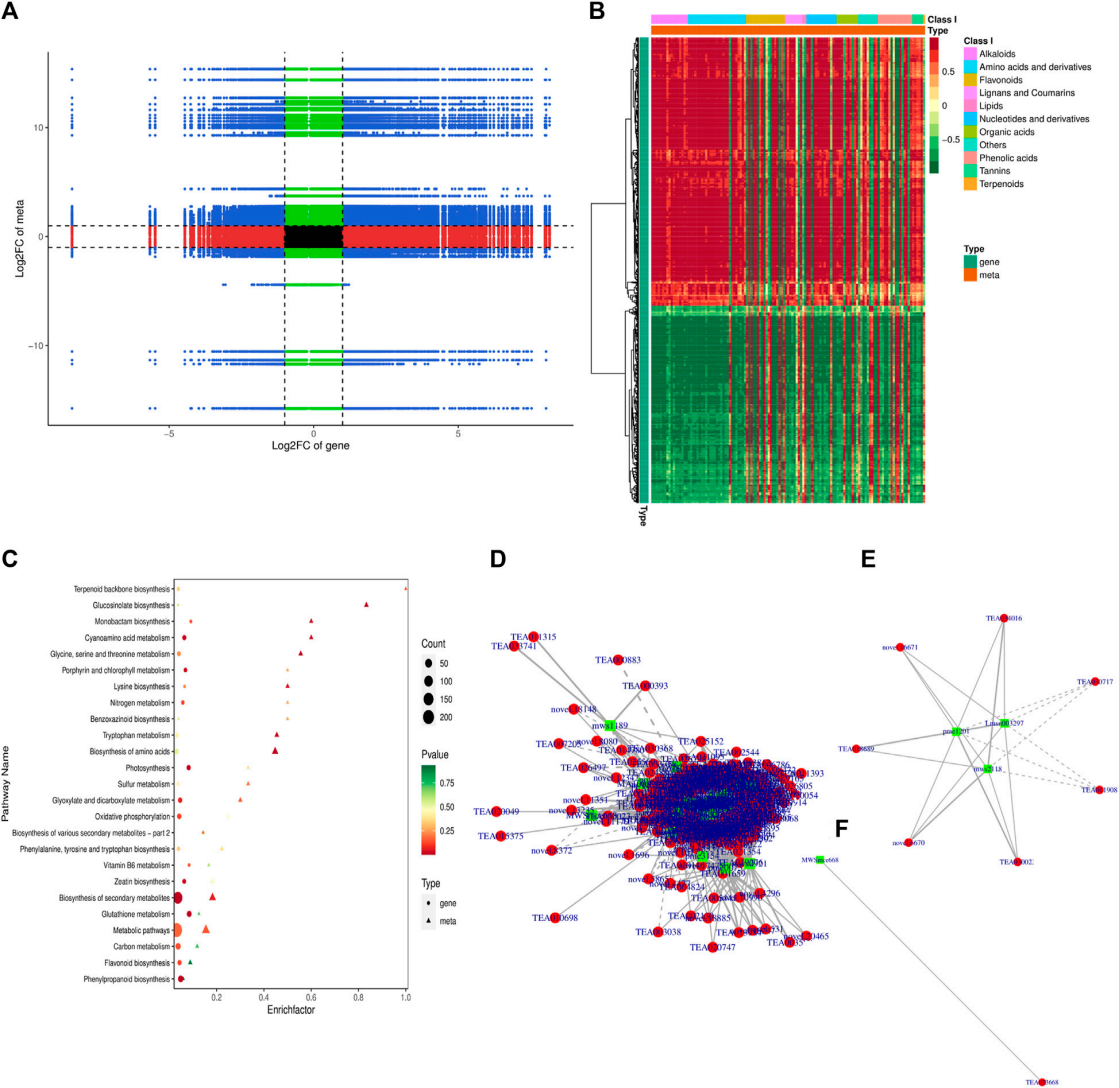
Conjoint analysis of transcriptome and metabolome **(A)** Nine-quadrant diagram of metabolite and transcriptome expression patterns **(B)** Correlation heatmap of metabolite and transcriptome expression patterns **(C)** KEGG pathways associated with co regulated DAMs and DEGs **(D)** correlation network of all the co-regulated DEGs and DAMs **(E)** correlation network of co-regulated DEGs and DAMs associated with flavonoid biosynthesis pathway **(F)** correlation network of co-regulated DEGs and DAMs associated with chlorophyll biosynthesis pathway.

A conjoint analysis of the transcriptome and metabolome was performed to investigate the relationship between genes and metabolites. KEGG pathway enrichment analysis was conducted to identify co-enriched pathways of differentially expressed genes (DEGs) and differentially accumulated metabolites (DAMs). The analysis revealed that DEGs and DAMs were enriched in 53 KEGG pathways, including biosynthesis of secondary metabolites, flavonoid biosynthesis, and biosynthesis of amino acids (Supplementary Table S5; Figure 6C). The Pearson correlation coefficient (PCC) was calculated to evaluate the correlation between DEGs and DAMs, and a nine-quadrant diagram was generated to show the fold changes of DEGs and DAMs with a PCC >0.8. The I and III quadrants showed a positive correlation between DEGs and DAMs, while the VII and IX quadrants showed a negative correlation (Figure 6A). A clustered heatmap was constructed to visualize the DAMs related to DEGs, which were classified into several categories, with alkaloids, amino acids, and flavonoids being the largest categories (Figure 6B).

We further explored the correlation and developed a network of all the co-regulated DEGs and DAMs (Figure 6D). Furthermore, each KEGG pathway and its associated DEGs and DAMs were explored to understand the regulatory relationship of DEGs and DAMs. We focused on flavonoid biosynthesis and photosynthesis pathways (Figures 6E, F). In the flavonoid biosynthesis pathway, our study identified three coregulated metabolites, namely, pme1201 (Phloretin), Lmsn003297 (3,4,2′,4′,6′-Pentahydroxychalcone-4′-O-glucoside), and mws2118 (Phloretin-2′-O-glucoside (Phlorizin)), which were influenced by 12 differentially expressed genes (DEGs) including *FL3H, PKSB, FAP3, VSR1, JOX1, ANRCH, SAT, BEATH, VINSY, SAT,* and *ANR* with one unknown gene (Supplementary Table S5). Among these DEGs, *FAP3*, *ANR*, *FL3H*, *ANRCH* and *VSR1* displayed a significant positive correlation with the three metabolites, while *PKSB*, *SAT* showed a significant negative correlation with their accumulation pattern (Figure 6E). In contrast, we only identified one metabolite, MWSmce668 (ATP; Adenosine 5′-Triphosphate), positively associated with TEA023668 (*PSBY*; *Photosystem II core complex proteins*) in the photosynthesis pathway (Figure 6F). Nonetheless, we also identified *YCX91, ATPB, FER1, PSAEB, PSAK, PSAL, PSAN, PSBC, PSBW,* and *PSB28* with one unknown gen as DEGs in the photosynthesis pathways (Supplementary Table S5).

### qRT_PCR based verification of expression profile of candidate genes

**FIGURE 7:**
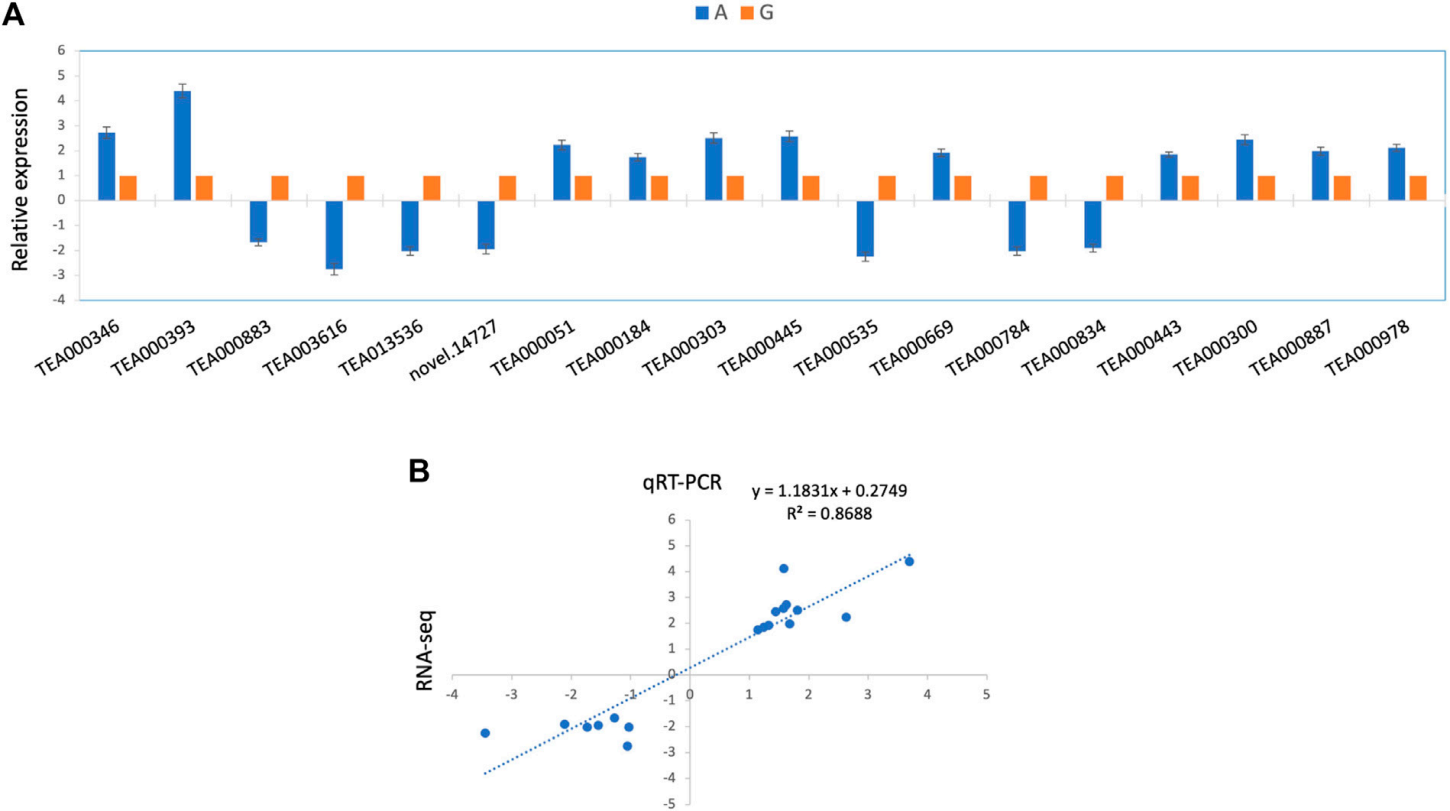
qRT-PCR based expression analysis of selected genes **(A)** Relative expression profile of 17 selected genes based on qRT-PCR **(B)** Correlation estimates of RNA-seq quantitation and qRT-PCR results.

To understand the regulation patterns of candidate genes in two contrasting genotypes (G and A), we conducted qRT-PCR analysis for all the candidate genes, using the primer sequences provided in Supplementary Table S6. The expression profile of the genotype was considerably different among two genotypes (Figure 6A). Among 18 candidate genes, 11 depicted positive expression patterns in albino leaves (A), while seven showed negative expression patterns (Figure 7A). Furthermore, the qRT-PCR results also support the observed variations in the whole transcriptomic datasets. Notably, a significant positive correlation (r^2^ = 0.87) was observed between RNA-seq and qRT-PCR data (Figure 7B). This finding provides further support for the reliability and accuracy of the qRT-PCR method for assessing gene expression levels in different genotypes.

## Discussion

We systematically utilized transcriptomics and metabolomics in the present study to understand the molecular mechanisms underlying albinism in Yanling Yinbiancha by comparing albino and green leaves. A significant difference in chlorophyll contents and carotenoids were identified in both leaves, which is consistent with previous studies reporting a lack of chlorophyll and an increase in carotenoids due to albinism (Li et al., 2016; Zheng et al., 2021; Liu et al., 2022). The biosynthesis and degradation of chloroplasts involve a complex cascade of enzymatic reactions within chloroplasts. Any aberration, such as genetic mutations or alterations in gene expression, in this intricate process, can disturb the normal metabolism of chlorophyll, leading to variations in leaf pigmentation in plants (Rüdiger, 1997). Albino tea cultivars exhibit altered chemical composition in leaves, which affects both the sensory quality and health benefits of tea (Du et al., 2006). Our study identified differential metabolites between albino and green leaves, with many enriched in amino acid metabolic pathways. Specifically, previous studies have shown a positive correlation between decreased chlorophyll an and chlorophyll b contents and increased total amino acid contents in albino tea plant cultivars (Feng et al., 2014; Wang et al., 2016). These findings suggest that the alteration of chlorophyll contents in albino tea plants may lead to changes in amino acid metabolism, ultimately affecting the composition of tea leaves and their associated health benefits.

Our analysis identified several differentially expressed genes (DEGs) involved in chlorophyll biosynthesis, suggesting that these genes may influence the production of chlorophylls in albino leaves. Chlorophyll and carotenoid pigments are synthesized from isopentenyl diphosphate (IPP) and its isomer dimethylallyl diphosphate (DMAPP) via the mevalonic acid (MVA) and the 2-C-methyl-d-erythritol 4-phosphate (MEP) pathway (Paniagua-Michel et al., 2012; Tetali, 2019). In Arabidopsis, *DXS* (*1-deoxy-D-xylulose-5-phosphate*) mutants, which exhibit a distinct albino phenotype with reduced chlorophyll and carotenoid levels, provide evidence for the role of the MEP pathway in pigment synthesis (Rodríguez-Villalón et al., 2009). Zheng et al. (2021), identified *CsDXS* in *C. sinensis* with suppressed expression in albino leaves and suggested influencing chlorophyll levels. Another study by Li et al. (2019), identified *DXS1* and *DXS2* with suppressed expression resulting in pigment deficiency in “Xiaoxueya” tea cultivar. Moreover, impact of *DXS* gene expression on chlorophyll and carotenoid levels has been demonstrated in both tomato and Arabidopsis. A tomato mutant, wls-2297, which experiences a 38.6-kb deletion due to a single T-DNA insertion affecting the DXS1 gene, exhibited a significant reduction in both chlorophylls and carotenoids (García-Alcázar et al., 2017). In contrast, over-expression of DXS in transgenic Arabidopsis led to an increase in both chlorophylls and carotenoids (Estévez et al., 2001). We identified several DEGs involved in chlorophyll biosynthesis, such as *CLH2*, *CB4A*, *CB24*, *SGR*, *PSBC*, *AB3C*, *ARR1*, *AB3C*, *HEM11*, *SUOX*, *CRD1*, and *CIP7*.

*CB4A* is a gene found in plants that have been shown to play a crucial role in chlorophyll synthesis. It encodes a magnesium chelatase subunit, which is involved in the first step of the chlorophyll biosynthesis pathway. Specifically, the *CB4A* protein is responsible for the insertion of magnesium into protoporphyrin IX, which is a key step in forming chlorophyll molecules. Loss-of-function mutations in *CB4A* have been shown to result in a severe reduction in chlorophyll levels and a corresponding decrease in photosynthetic efficiency (Masuda and Takamiya, 2004; Zhang et al., 2006). Therefore, the *CB4A* gene is an important target for research aimed at improving the yield and quality of crops through the optimization of chlorophyll synthesis.

The *Stay Green* (*SGR*) gene is a plant-specific nuclear gene involved in chlorophyll degradation during leaf senescence. Recent studies have shown that *SGR* also plays a role in regulating chlorophyll biosynthesis, particularly in the synthesis of chlorophyll b (Barry, 2009). SGR gene mutants in Arabidopsis and rice have been shown to have reduced levels of chlorophyll and delayed leaf senescence (Park et al., 2007; Barry, 2009; Lu et al., 2017). These mutants also showed reduced expression levels of genes involved in chlorophyll biosynthesis, including *POR* (*Protochlorophyllide oxidoreductase*), *CAO* (*Chlorophyllide a oxygenase*), and *CHLH* (*Mg-chelatase subunit H*). Further studies have revealed that SGR interacts with HEMERA, a transcription factor involved in chlorophyll biosynthesis, to regulate the expression of these genes (Jiang et al., 2007). Overall, these findings suggest that the SGR gene plays an important role in the regulation of both chlorophyll degradation and biosynthesis in plants. Ma et al. (2018), also suggested potential role of *SGR* genes along with other Chlorophyll biosynthesis-related genes to play significant role in albinism in tea plants. Moreover, the *PSBC* gene encodes for the PSII reaction center protein. It is involved in assembling the PSII complex, which is important for the light-dependent reactions of photosynthesis and, ultimately, the synthesis of chlorophyll (Zhang et al., 2011; Yamamoto, 2016).

Transcription factors (TFs) are proteins that bind to DNA and regulate gene expression by either activating or repressing transcription. They play a crucial role in various biological processes, including chlorophyll biosynthesis. We identified 105 TFs differentially regulated in albino leaves compared to green leaves. Several studies have reported the roles of transcription factors in regulating the expression of genes involved in chlorophyll biosynthesis. For instance, the *AP2/EREBP* family transcription factor *OsEREBP1* was found to regulate gene expression in the biosynthesis of carotenoids and chlorophylls in rice (Dang et al., 2013). The bZIP transcription factor HY5 was shown to regulate the expression of genes involved in the biosynthesis of carotenoids and chlorophylls in Arabidopsis (Zhou et al., 2015). The MYB transcription factor CsMYB60 was reported to regulate the expression of genes involved in the biosynthesis of flavonoids, which play a role in the biosynthesis of chlorophyll in cucumber (Chen et al., 2021). The *WRKY* transcription factor *AtWRKY75* was found to regulate the expression of genes involved in the biosynthesis of chlorophyll and the response to abiotic stress in Arabidopsis (Chen et al., 2010). These studies demonstrate the crucial role of transcription factors in regulating the expression of genes involved in chlorophyll biosynthesis. Further functional verification in *C. sinensis* can yield significant insight into their role in albinism.

Albinism is a genetic disorder in plants that affects the production of chlorophyll and other pigments, leading to the absence osf green coloration in leaves and other plant tissues. Flavonoids have been shown to be involved in the regulation of pigment synthesis in plants. In Arabidopsis, several genes involved in flavonoid biosynthesis, such as *CHS*, *CHI*, and *F3H*, have been found to be upregulated in albino mutants, suggesting a potential role for flavonoids in compensating for the loss of chlorophyll pigments (Lichtenthaler and Buschmann, 2001). In this study, we identified *ANR* (*Anthocyanidin reductase*) and *ARR1* (*Arabidopsis Response Regulator 1*) genes with upregulated expression patterns, suggesting their involvement in increased biosynthesis of flavonoids in albino leaves, which was also confirmed through metabolome quantification. Our results confirmed the previous findings of up-accumulation of flavonoids in albino plants (Xiong et al., 2013; Sun et al., 2022).

Together, the study provides significant insight into the differences in transcriptome and metabolome of albino and green leaves of Yanling Yinbiancha. We identified several candidate genes associated with chlorophyll biosynthesis, carotenoid biosynthesis, and stay green trait with co-regulated metabolites. The genes related to albinism identified in this study would be important targets for genetic modification or molecular markers development, which will be critical for improving the cultivation efficiency of *C. sinensis* and provide further scope for strengthening the existing germplasm and utilization in breeding programs.

## Data Availability

The datasets presented in this study can be found in online repositories. The names of the repository/repositories and accession number(s) can be found below: NCBI BioProject (https://www.ncbi.nlm.nih.gov/bioproject/), PRJNA991710.

## Supplementary material

SUPPLEMENTARY FIGURE S1 Principal component analysis for transcriptome and metabolome datasets.

SUPPLEMENTARY FIGURE S2 Heatmap illustrating FPKM values of differentially expressed genes (DEGs).
