# Supplementary figures and images for: Insights into the physiological, molecular, and genetic regulators of albinism in Camellia sinensis leaves
Source: Front Genet. 2023 Sep 6;14:1219335. doi: 10.3389/fgene.2023.1219335 (PMC10516542; doi:10.3389/fgene.2023.1219335)

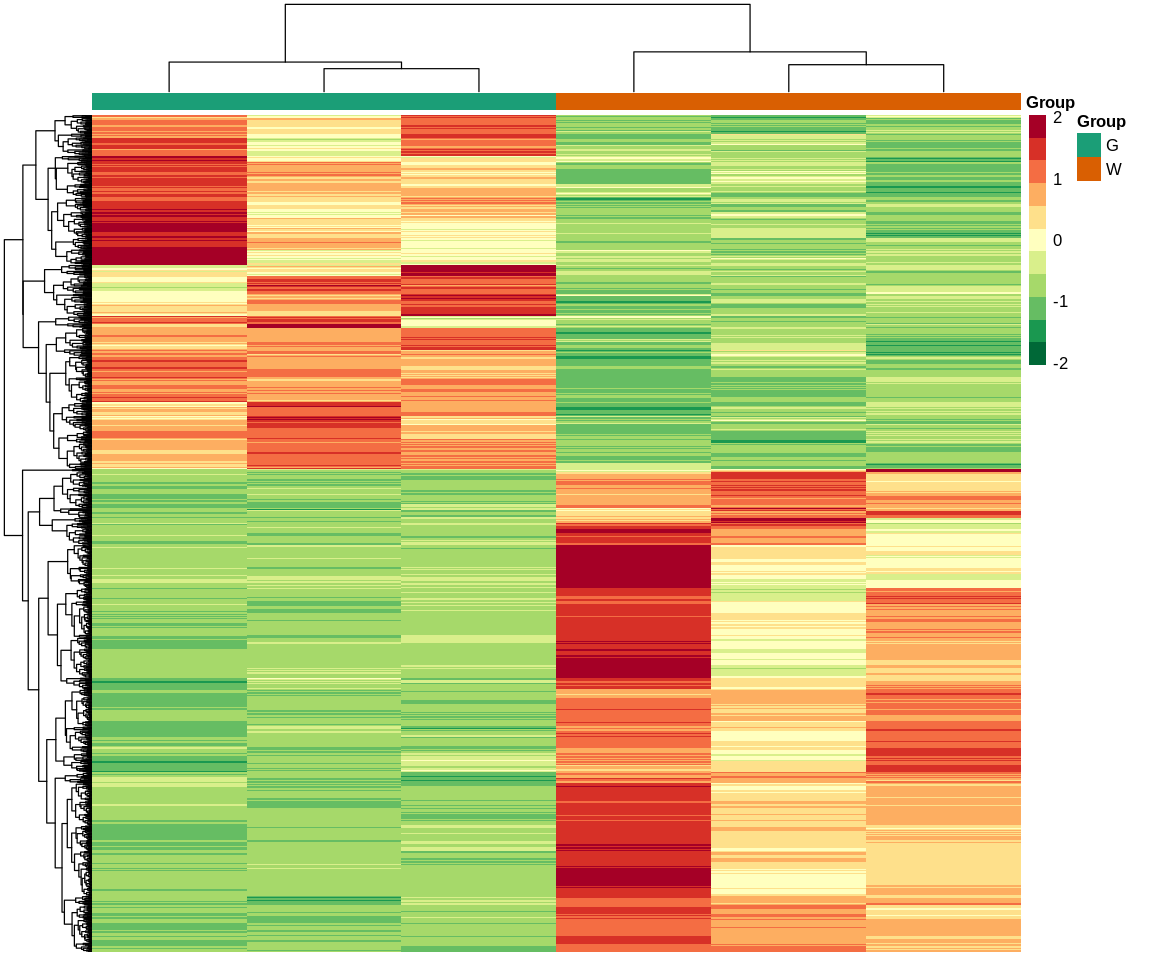

Supplement: Supplementary file 5 [file Image2.png]

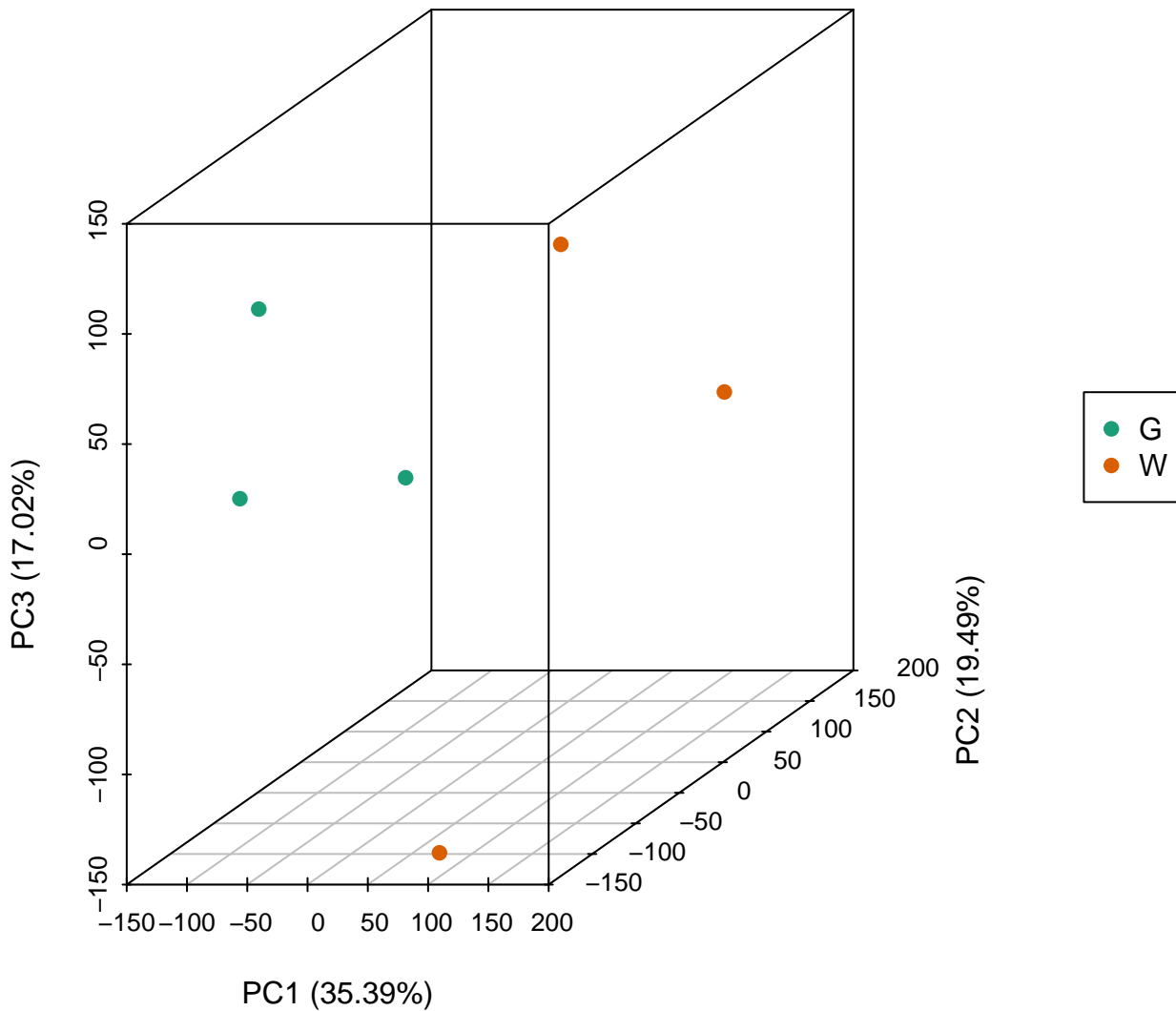

Supplement: Supplementary file 8 [file Image1.pdf]
